# Improving sleep health management in primary care: A potential role for community nurses?

**DOI:** 10.1111/jan.15577

**Published:** 2023-02-09

**Authors:** Mariam M. Basheti, Zeeta Bawa, Ronald Grunstein, Nicole Grivell, Bandana Saini, Christopher J. Gordon

**Affiliations:** ^1^ School of Pharmacy, Faculty of Medicine and Health The University of Sydney Camperdown New South Wales Australia; ^2^ Sleep and Circadian Research Group Woolcock Institute of Medical Research Sydney New South Wales Australia; ^3^ Brain and Mind Centre, School of Psychology, Faculty of Science The University of Sydney Camperdown New South Wales Australia; ^4^ Lambert Initiative for Cannabinoid Therapeutics The University of Sydney Camperdown New South Wales Australia; ^5^ School of Medicine, Faculty of Medicine and Health The University of Sydney Camperdown New South Wales Australia; ^6^ Royal Prince Alfred Hospital Sydney New South Wales Australia; ^7^ Adelaide Institute for Sleep Health/FHMRI Sleep, College of Medicine and Public Health Flinders University Adelaide South Australia Australia; ^8^ Susan Wakil School of Nursing and Midwifery, Faculty of Medicine and Health The University of Sydney Camperdown New South Wales Australia

**Keywords:** chronic illness, community nursing, insomnia, nurse ‐ patient interaction, nurse education, sleep health, workforce issues

## Abstract

**Aims:**

To explore community nurses sleep health practices and their perspectives on improving sleep health care provision.

**Design:**

An exploratory study utilizing the qualitative description methodology.

**Methods:**

Semi‐structured interviews were conducted with community nurses from May 2019 – October 2021. Interviews were audio‐recorded, transcribed, and subjected to an inductive thematic analysis using a constructivist–interpretive paradigm.

**Results:**

Twenty‐three Australian community nurses were interviewed. Participants frequently encountered sleep disturbances/disorders in their patients. Data analysis yielded three main themes: (1) Sleep health in the community serviced, (2) sleep health awareness and management, and (3) community nurses' A to Z of improving sleep health. The most common sleep disorder presentations were insomnia and sleep apnea. Although most community sleep apnea cases were appropriately managed, insomnia was often mismanaged. Participants described their sleep health knowledge as deficient, with the majority advocating for increased sleep‐related education tailored to their profession. Other important factors needed for improving sleep health provision were standardized patient treatment/referral pathways, increased interprofessional collaboration, and sufficient time for patient consults.

**Conclusion:**

Community nurses service a patient population that requires increased sleep health care. However, they are currently underequipped to do so, leading to suboptimal treatment provision. Providing community nurses with the appropriate resources, such as increased sleep‐related education and standardized treatment frameworks, could enable them to better manage sleep disturbance/disorder presentations, such as insomnia.

**Impact:**

Little is known about how community nurses care for patients with sleep disturbance/sleep disorders. This study found that contemporary sleep health care was lacking due to knowledge deficits, competing challenges, and a need for standardized care pathways. These findings can inform the development of targeted education/training and standardized guidelines for community nurses providing sleep health care to patients as well as the design of future practice models of care provision.

**Patient or Public Contribution:**

Previous research by authors has involved extensive engagement with patients and health professionals, such as community pharmacists, general practitioners, and naturopaths who play a role in sleep health in the primary health care sector. These previous research projects built a significant understanding of the patient and health practitioner experience and have provided the background to the concept and design of this study.

## 1 INTRODUCTION

Poor sleep health is a global issue and is associated with physical and mental health comorbidities (Colten & Altevogt, 2006). Improving sleep health is now recognized as a key contributor to optimal health and wellbeing. However, in clinical practice, poor sleep health is often unrecognized or overlooked in preference to other health conditions (Hale et al., 2020). Community nurses are at the forefront of patient care and commonly care for a diverse patient population, typically with a high prevalence of chronic diseases, especially in older people. Accordingly, community nurses are likely to encounter patients with poor sleep health and sleep disorders, which may impact on the patient's overall health and recovery from illness. As community nurses have a high degree of practice autonomy, they are uniquely placed to assess and manage patient's poor sleep to provide holistic care. As the clinical importance of sleep health continues to develop, it is essential to understand and delineate the roles that community nurses perform and ultimately facilitate improvements in patients' sleep health.

## 2 BACKGROUND

Community nurses have an integral role in primary health care with diverse roles such as providing health education and promotion through to complex chronic disease management. In Australia, nurses do not require specific educational requirements for community nursing; however, some may have postgraduate qualifications and/or specialized training in certain areas of care. Community nursing in Australia can be classified as nursing practice conducted outside of tertiary healthcare settings, such as the patient's home, primary care health settings, community health centres, or pharmacies and clinics. In recent years, there has been an increased reliance on community nurses driven by increases in the aging population and increased chronic disease prevalence coupled with the need to shorten length of stay in hospitals for patients who can receive care in the community or at home (Blay et al., 2022; Kerkstra & Vorst‐Thijssen, 1991). Accordingly, patient illness acuity, disease complexity, and multimorbidity has increased, and community nurses need to be able to address these increasingly complex patient health needs efficiently. Community nurses are described as providing an “interpretative bridge” between the acute tertiary sector and community services (Australian Primary Health Care Nurses Association, 2022). Given the downstream sequelae and heavy costs that sleep disturbances/disorders, such as insomnia, impose on the health care system (Deloitte, 2011), a focus on exploring increased investment in preventive health interventions that can be delivered by community nurses is warranted.

Sleep health is one such aspect of lifestyle behaviors, fundamental to optimal physical and mental health, that underpins many chronic illnesses (Worley, 2018). The importance of sleep health on overall wellbeing has been recognized and strongly recommended as an essential health priority (Scott et al., 2021). The most common sleep disorders impinging on sleep health are insomnia and obstructive sleep apnea (OSA), with insomnia recording a global prevalence of up to 30 percent and OSA affecting approximately 1 billion adults around the world (Bartlett et al., 2008; Benjafield et al., 2019; Bhaskar et al., 2016). Disturbed sleep can have severe effects on brain health, immune response, and cardiometabolic and mental health (Dikeos & Georgantopoulos, 2011). In addition, sleep disorders, such as insomnia and OSA, are highly prevalent but under‐recognized and under‐reported (Almeneessier et al., 2018; Culpepper, 2005; Motamedi et al., 2009). Downstream consequences of conditions such as insomnia and OSA include onset or progression of cardiometabolic disease, mental health disorders, and neurodegenerative conditions, making sleep health a target for prevention‐based activities (Colten & Altevogt, 2006; LeBlanc et al., 2018; Lin et al., 2018; Medic et al., 2017; Rosenberg, 2021; Yeghiazarians et al., 2021). For instance, evidence indicates that patients with diabetes or those experiencing depression are more likely to develop insomnia (Khandelwal et al., 2017), and patients with heart disease or chronic obstructive pulmonary disease have an increased risk of developing OSA (Hamilton & Joosten, 2017). Moreover, medications commonly used to treat these comorbidities in community settings increase the risk of or exacerbate sleep disorders, such as insomnia. For example, beta blockers, angiotensin‐converting enzyme inhibitors, and selective serotonin reuptake inhibitors are known to increase adverse effects on sleep (Neel Jr, 2013). It is also important to note that the presence of these sleep disorders exacerbates these diseases (LeBlanc et al., 2018; Lin et al., 2018; Rosenberg, 2021; Yeghiazarians et al., 2021).

Sleep health problems, despite a highly frequent presentation, are often not recognized or relegated in prioritization of care by primary care health professionals such as general practitioners (GPs) (Sake et al., 2019). Models of care incorporating practice nurses and other allied health professionals such as pharmacists, in case detection, triage, and treatment support pathways, have been demonstrated (Chai‐Coetzer et al., 2013; Hanes et al., 2015). Community nurses frequently provide care for patients with a multitude of conditions and illnesses, focusing on their health care needs, with a strong likelihood of encountering patients with disturbed sleep or sleep disorders. However, there is currently insufficient research showcasing the community nurse role in sleep health management and care provision in their patient populations. Accordingly, this study will document the community nurse experience in sleep health care and inform potential future practice change.

## 3 STUDY METHODS

### 3.1 Aims

The aim of the study was to explore current sleep health practices carried out by community nurses and to gauge their perspectives and ideas around the change needed to achieve improved community nurse‐delivered sleep health care.

### 3.2 Design

Given the exploratory aims of the study, a qualitative descriptive study was conducted (Doyle et al., 2020; Sandelowski, 2010), using semi‐structured, one‐on‐one interviews as the data collection method. A qualitative descriptive method is well‐suited for research that aims to gauge the experience of participants in cases when little is currently known about the phenomenon being explored. Qualitative description allows for the findings from the data to be presented as a richly descriptive summary that fits the information captured closely. This method does not require complex inferential steps, for example, those required in the phenomenology or grounded theory methods (Colorafi & Evans, 2016; Sandelowski, 2010). The qualitative description method also allows for choices in terms of sampling, data collection, and use or non‐use of frameworks or theories in the analytical process (Colorafi & Evans, 2016). The semi‐structured interview format is most often used when background information about a specific topic from key informants, or an institutional perspective, is required. This interview format allows a deep dive into the thoughts, feelings, and attitudes of interviewees about a particular topic (DeJonckheere & Vaughn, 2019). As Australian community nurses sleep health management practices or future support needs have not been previously explored, a qualitative descriptive method utilizing semi‐structured interviews to collect data was deemed suitable for this study (Bradshaw et al., 2017; Doyle et al., 2020). The study was conducted in New South Wales, Australia; however, there were no restrictions on participant location given that interviews were conducted via telephone.

### 3.3 Participants

In a convenience‐based sampling approach, potential participants were initially identified through professional networks of the research team through email contact explaining the purpose of the project. Participants who were community nurses currently working in community settings were included in the study. All participants had completed a Bachelor of Nursing degree followed by national registration with the Australian Health Practitioner Regulation Agency (AHPRA) to practice as a registered nurse in Australia. Nurses working in community pharmacies were also included as they were providing primary health care in the community and will be highlighted as such throughout the manuscript wherever applicable. Nurses working in general practice settings were not included in this study, as these registered nurses have different roles and patient contact from community nurses. In addition to initial email contact, passive snowball sampling methods were used to increase participant recruitment, whereby contacted participants were encouraged to pass details of the research study/contact details to potentially interested colleagues, who could then contact the research team directly for study participation (Parker et al., 2019). Consent was obtained in written and verbal form prior to interviews. All participants were provided with a $50 gift voucher following the interview. Participant recruitment was governed by thematic saturation (i.e., recruitment continued until no new insights or ideas were emerging from the data; Ando et al., 2014).

### 3.4 Data collection

Qualitative semi‐structured interviews were conducted with a maximally varied, convenience‐based sample of community nurses from May 2019–October 2021 (data collection was temporarily halted during COVID‐19 lockdowns). Interviews were conducted via telephone by two research team members (MB; female, pharmacist, PhD student and ZB; female, pharmacist, PhD student). Interviews were carried out using a cognitively funnelled interview guide (Appendix: Table 1A), i.e., starting off with basic demographic questions before moving onto more cognitively challenging queries that require increased thought process and discussion (Rosala & Moran, 2022). The semi‐structured interview guide was informed by an extensive review of the literature and the practice expertise of the research team (Cheung et al., 2014; Espie, 2009; Meaklim et al., 2020; Sake et al., 2018, 2019). Prompts were used if required to guide the interview (e.g., when participants requested more explanation to better understand what the question was aiming to look at). Probes were also used wherever appropriate to help researchers gauge the “full picture” and gain a deeper understanding of the matters discussed during the interview. Interviewers were provided training by a senior nurse researcher (CG) to enhance interview conduction. All interview recordings were transcribed verbatim, and transcripts were reviewed against audio recordings to ensure accuracy.

### 3.5 Ethical considerations

University research ethics committee approval for study conduct was obtained prior to commencement (project number: H‐2018‐257). Fundamental principles of ethical practice, such as providing participants with a study information sheet, obtaining participant consent, and ensuring confidentiality and voluntary participation/freedom of study withdrawal at any time, were followed through.

### 3.6 Data analysis

All participants were coded as a pseudonym during interview discussions to maintain deidentification during transcript coding, and a unique de‐identification code (CN‐XX) was allocated to participant interviews. Four members of the research team (MB; primary researcher, ZB; co‐researcher, CG and BS; senior researchers) independently coded 10% of the data and had team discussions until consensus around coding was reached. Following that, the primary researcher (MB) went on to code the remaining data and had regular discussions with the senior researchers (CG and BS) regarding thematic content. All transcripts were uploaded to NVivo 1.5 and subjected to a six‐phase inductive thematic analysis (from data familiarization through to coding, thematic search, thematic review, naming the themes, and writing up the results; Braun & Clarke, 2006). Data findings were reported through a constructivist–interpretive paradigm (building meaning through interpretation of participants lived experiences; Mackenzie & Knipe, 2006).

### 3.7 Validity and study rigour

Trustworthiness was established in all phases of our qualitative research using set criteria; Credibility, Transferability, Dependability, and Conformability (Nowell et al., 2017). Details of the strategies used to achieve these set criteria are highlighted in Table 1. Further, the consolidated criteria for reporting qualitative research (COREQ) checklist was adhered to wherever applicable and provided in the Appendix (Tong et al., 2007).

**TABLE 1:** Trustworthiness checklist.

Criteria	Strategy utilized	Process of achievement
Credibility	*Researcher triangulation*	Team discussions around codes and thematic structure until consensus reached.
	*Data triangulation*	Field notes were taken during interviews and used during data analysis where appropriate.
	*Peer debriefing*	Conducting regular primary and senior researcher debriefs regarding thematic deduction and content.
Transferability	*Maximally varied participant sample*	Allowing for a broad representation and consequently for credible conclusions to be drawn.
	*Detailed study description*	Detailed description of the methodology, data collection, and data analysis process to achieve optimal repeatability and transferability.
Dependability	*Independent review of data*	Primary and senior researchers discussed independent data analysis results until consensus was reached.
Conformability	*Checking context*	Codes were reviewed for context to ensure accurate data interpretation and presentation.
	*Reflexivity*	Including researchers from different health backgrounds allowed for reflexivity in data analysis and presentation.
	*Presenting data derivations*	Participant exemplar quotes were provided to support the results reported.
	*Maintaining audit trails*	Making NVivo audit trails (created by primary researcher) available to other research team members for cross‐checks.

## 4 FINDINGS

All participants (*n* = 23) were registered nurses and currently working in community settings, with a few specifically working in community pharmacy (CN01 and CN03). Participant demographics are highlighted in Table 2.

**TABLE 2:** Participant demographics.

Variables	(*n* = 23) (%)^a^
Gender	
Female	22 (96)
Age (years)	
25–34	4 (17)
35–44	2 (9)
45–54	13 (57)
55–64	3 (13)
75–84	1 (4)
Location	
Regional	1 (4)
Metropolitan	21 (91)
Rural	1 (4)
Highest professional qualifications	
Graduate certificate	5 (22)
Diploma	2 (9)
Bachelor's degree	12 (52)
PhD/Master's degree	4 (17)
Community nursing experience (years)	
<1–5	9 (39)
6–10	7 (31)
11–20	6 (26)
>50	1 (4)

*Note*: *PhD* Doctor of Philosophy.

^a^ Rounded to the most appropriate whole number.

Semi‐structured qualitative interviews were conducted, ranging in length from 17–43 min (average 30 min). All participants were involved in the direct care of patients (most commonly in older age groups with significant comorbidity). Generally, these comorbidities led to patients requiring individualized care, for example, patients with a life‐limiting illness needing palliative care and those with cardiovascular, endocrine, or respiratory disease requiring standard condition‐specific care and specialized care for the complications that arise, such as catheter placement or wound care. Interestingly, sleep disorders/disturbances were common in the patients cared for by community nurses with the majority of participants stating that they frequently encountered patients with sleep‐related concerns.

In‐depth analysis of the data yielded three main themes: (1) *Sleep health in the community serviced*, (2) *Sleep health awareness and management*, and (3) *Community nurses' A to Z of improving sleep health*.

### 4.1 Theme 1: Sleep health in the community serviced

In caring for patients in a range of settings and across a spectra of health conditions, most participants suggested that these health issues and the medications used to treat them were often the source of patients' sleep complaints. For example, it was described that patients with cardiovascular or renal problems would complain about having to wake up during the night due to episodes of breathlessness or urinary urgency and find it difficult to fall back asleep afterwards. A quote typical of the disease‐related sleep impairments that participants encountered was

Nocturia, quite frequently in a lot of men and women, getting up multiple times overnight, disturbing their sleep or leading to a poor sleep pattern as a result of urinary function or dysfunction. – CN15

Sleep disorders such as insomnia and OSA were also frequently reported, with most participants describing insomnia presentations as either a ‘standalone’ complaint or as secondary to another comorbid condition. Participants mentioned that patients with insomnia also expressed increased anxiety about their health. As opposed to OSA, which was usually previously diagnosed and actively treated, participants stated that most of the longstanding insomnia presentations were not diagnosed, and some patients would self‐medicate or simply dismiss the issue as something they have to ‘live with’.

I think in my practice, a lot of people suffer from insomnia and whether that's due to their age, or I suppose other medical factors that's generally what people complain to me about, that they can't sleep. Yeah. Or they only have very short amounts of sleep and then they wake up and can't get back to sleep. – CN14

OSA was most commonly reported by the community nurses working in community pharmacies or community nurses with respiratory or cardiovascular patients, mainly due to the comorbidity aspect of OSA. Community nurses reported that the majority of OSA patients were prescribed a continuous positive airway pressure (CPAP) device. Other sleep disorders, such as restless legs syndrome and hypersomnia, were mentioned infrequently.

### 4.2 Theme 2: Sleep health awareness and management

#### 4.2.1 Subtheme 2.1: Sleep health knowledge

Whilst participants acknowledged the importance of sleep on whole body health, interestingly, the majority described their sleep health knowledge as deficient, with few previous encounters of sleep education or training. They also described that this knowledge gap impacted negatively on their ability and confidence to appropriately manage patients’ sleep health problems.

I would say that I'm not (confident in sleep health management) – I would probably only give a small amount of advice. I've not personally been educated well enough in that area to feel that I could support someone fully. – CN12

#### 4.2.2 Subtheme 2.2: Management approach

Although participants had a basic understanding of sleep disorders, the majority did not proactively link symptomatic cues to potential sleep disorders and would generally manage the patient on a symptomatic‐relief basis. Participants usually instigated a discussion with patients who complained about their sleep. Some participants, such as those caring for palliative care patients or patients with OSA, also had routine sleep health questions that they used with patients during clinical visits, but this was not common.

So, we discuss sleep every visit with the patient. We use a PCOC (palliative care assessment tool) for the severity of their symptoms. So sleep is top of the list and we ask them how they sleep, if they need anything to help them sleep, what helps them sleep or what strategies do they put in place. We ask them if their difficulty in sleeping causes them distress. So, we ask that at every patient visit, and we will discuss that with the team. – CN21

If a sleep health problem was identified, participants stated that they would assess patients sleep patterns, sleep habits, type of sleep disturbance, impact on activities of daily living, and what measures patients took to address their sleep issue. They would undertake a detailed symptomatic and medication history to determine possible aetiology of the sleep problem. Some participants stated that they used sleep diaries to better understand patients' sleep patterns.

So I would do an assessment on the signs and symptoms that they're experiencing relating to the sleep disorder, so I would be looking at the duration of how long it has been an issue, how many hours of sleep they're getting, what's preventing them from sleep or if they do fall asleep, if they wake up intermittently during the night and what's causing that. (…) So yes, I would ask them to do a sleep diary for a week and write down, you know, when they're falling asleep, when they're waking up, how many hours they're getting, are there certain things that are making it more difficult to sleep, and I get them to write all that down, and then we could look at that the next week and then take it from there. – CN04

After assessing the patient, participants used various management approaches, depending on the individual patient case, their sleep concern, and the participants’ own knowledge and expertise with sleep disturbances/disorders. When it came to OSA, participants who were experienced with its management were able to help diagnosed patients troubleshoot CPAP use and refer potential OSA patients to their GP/specialist for a sleep test (assessment of the patient's sleep) if needed. However, in the case of insomnia and other sleep disturbances, most participants used an “ad hoc” management approach; sleep hygiene education, complementary/over the counter (OTC) medicine suggestions, and GP referral and prescription medication recommendations as the most common ways to manage these patients. Very few participants recommended behavioural strategies, such as stimulus control, to their patients, and only a small number of participants would also recommend tapering benzodiazepines to patients who have been taking them long‐term.

I mean, I sometimes would go down the route of suggesting, if it's an ongoing issue, maybe see your GP, maybe see if you could get some form of either sedation or something you can get over the counter at a pharmacy to get you back into your normal sleep routine should – as a temporary basis. – CN07

If I was having a consultation with a patient and it appears to me that they could well have sleep apnea that has not been diagnosed I would very strongly recommend them on to see somebody to have further discussion about that and have that assessed more thoroughly. – CN03

### 4.3 Theme 3: Community nurses' A to Z of improving sleep health

#### 4.3.1 Subtheme 3.1: Importance of role and expanding their scope

Participants regarded their community role conducive to sleep health care, stating that their ability to spend uninterrupted time with their patients, consult them in the comfort of their own homes, and see them on a long‐term basis all meant that they could build trusting relationships. Some participants also mentioned that their mode of health provision could also allow them to request to see consenting patients' bedrooms/sleep environments and make appropriate recommendations for adjustments. Participants further explained that targeted training and education would help propel any potential future sleep health roles they may take up.

I think, more specifically for community nurses, I mean, we do tend to develop long‐term relationships with our patients, because we're seeing them for chronic disease management and its complications, so we're seeing people for a really long time sometimes, so we definitely are in a good place to start discussions about all kinds of things. – CN10

Patient consultation, brief sleep hygiene education, product recommendation, and patient referrals were the main ways participants were currently involved in when it came to helping patients with sleep health issues. While only a few participants felt that providing sleep health care was not within their scope of practice, the majority believed that they could and should have greater involvement in this health area, given their frequent patient contact and autonomous practice. Participants also stated that they are usually the first point of contact for the specific patients they cared for regarding sleep concerns as they are able to have a casual, conversational consult where patients are able to open up about their “minor” health issues such as sleep.

I think generally nurses are the coalface of most interactions with most patients before – well, often before or during a process with the doctor or with anybody else that they might be speaking to, because I think the conversation with a nurse is a far less formal conversation than they would have with other health – than what they would have with other health professionals. – CN03

#### 4.3.2 Subtheme 3.2: Achieving greater sleep health involvement in community care

Participants further explained that they are well‐suited to provide sleep health care in various forms, including *initial screening* (being able to use standardized tools to formally assess the patient with a potential sleep disorder), *triage* (referring the patient on to more specialized care if needed), and finally *patient education*. They viewed triage as an integral role, given most perceived themselves as a point of liaison between the patient and the GP, and as having some knowledge about acceptable referral pathways in the health system for accelerating patients’ care. Patient education, however, was considered the most important role community nurses can play by most of the participants. It was expressed that having sufficient knowledge about sleep health and sleep disorders would allow participants to pass that on to their patients, which would ultimately improve sleep health awareness and management in the community.

Well certainly screening patients for sleep disorders and potential, I guess, basic management of that, or ability to refer to more experienced practitioners. – CN21

I think we could be more involved by giving a lot more education on sleep disorders, because I think there's a lot of people that perhaps don't realise how important it is or how many people have sleep disorders and that go untouched, uncovered. – CN07

All participants believed that in order to implement adequate change to current sleep health care regimens, some aspects had to be addressed. One issue that impacted patient care was a lower prioritization of sleep health by community nurses and other health professionals.

Some participants even suggested that lower sleep health awareness inculcated misperceptions around sleep, leading to inadequate patient care.

A lot of patients say that “when I say to my doctor I can't sleep, they just say it's part of aging”. So it's not like that. You need to have knowledge. Yes. It's a problem for them and making awareness for health professionals, that it is a main issue, and then teach that to the patient as well. – CN13

Yeah. How can you offer things when you don't know of them? I mean, everyone knows, you know, what ‐ the things you tell kids, I suppose, no devices and, you know, lower the lights. And you know, try and minimize distractions, the sort of, you know, that better way to get your body ready to sleep. Yeah. But beyond that, yeah. I don't know. – CN14

Another issue raised by participants was time and task pressures; they stated that they are usually time‐poor and would spend most of the consult discussing and caring for the patient's other comorbidities or acute care needs. Because of this, participants explained that sleep discussions tended to be brief, and the sleep concern managed cursorily rather than with a standardized evidence‐based approach.

I guess, our patient care is becoming increasing task orientated due to time constraints, increased number of patients with chronic illnesses, and I guess it would kind of – I believe it would take some more education to understand what this actually impacts upon and how important it is for us to monitor and manage it. – CN02

Furthermore, when it came to patient referral, most participants stated that clear referral pathways for higher orders of sleep health treatments are less well‐defined in the health system. Most mentioned if basic sleep hygiene advice/recommendations do not resolve the patient's issue, they provide either a *passive referral*, i.e., encourage the patient to speak to their GP or an *active referral*, where they would raise the issue with the GP themselves. However, most explained that the patient's sleep issue usually hits a “dead end” and not much gets done about it, leaving the patient to just “live with it”.

I think that's one of the main issues on that level. Like I don't, I like ‐ if I had a patient with a sleep problem, apart from me giving my very simple sort of advice or education, I don't know who I would turn to? – CN16

There were also key barriers highlighted in relation to patients and sleep health, such as their awareness and attitudes regarding the matter. Participants mentioned that most of their patients with a sleep disturbance would similarly trivialize the issue and sometimes refrain from bringing it up until proactively probed about it. Also, some patients were described as “indifferent” towards their sleep health as they expected to experience poor sleep given their morbid conditions or had simply lost hope in fixing their sleep given past unsuccessful attempts at doing so.

Particularly the patients that I've seen that have had chronic sleep issues, I think they've just lived with it and they go like ‘oh It's just the way it is.’ – CN22

#### 4.3.3 Subtheme 3.3: Overcoming the obstacles

Participants offered a range of possible solutions to counter these practice barriers. For example, the need for increased education and sleep health related training for community nurses was emphasized and various ways such as continuing professional development points or voluntary workshops suggested as delivery modes/formats.

Another important factor cited was collaboration, with participants stating that optimal care provision can be achieved with a multidisciplinary care team approach to sleep health management.

Further, participants expressed the need to develop standardized evidence‐based treatment guidelines and clear pathways for patient referral to specialized care, explaining that this could also aid in developing the much‐needed collaborative relationship between community nurses and other health professionals.

Finally, participants regarded “sufficient consult time” as an integral factor and also touched on the importance of utilizing some resources such as standardized assessment tools/sleep diaries to help disseminate improved sleep health care in the community.

If we are educated on the topic then we have a much bigger spectrum of knowledge to pass on to the patients and their families, and generally knowing what specialist should be involved, who we can contact, education around devices, education around other equipment or medications. So I think it really just does boil down to education of the nurses. – CN07

Look, I think it's really important that especially in the community, because it's generally a nursing – you know, it's generally just community nurses (providing direct patient care in the community), so I think it's really important to collaborate with other multidisciplinary health members, so with doctors, with physios, you know, OTs, dieticians. – CN10

## 5 DISCUSSION

To the best of our knowledge, this is the first research study that aimed to investigate how registered community nurses in Australia currently provide care for patients with sleep problems and sleep disorders and how they believe their sleep health care provision can be improved. Given the patient cohort they cared for, community nurses frequently encountered sleep disturbances and sleep disorders such as insomnia. Although common, participants were unable to provide optimal care for patients' sleep concerns and attributed this to multilayered issues ranging from their own and other health professional's insufficient knowledge/expertise in this health area, to lower overall community awareness of the salience of sleep in maintaining health, and to systemic issues such as lack of clarity in referral pathways, treatment guidelines, and clinical resources. The trivialisation of sleep health preceded sub‐optimal management, all the way across the health continuum ‐ from sleep disorder recognition, appropriate treatment provision, patient education, and specialized referral. Community nurse participants were positive about expanding their sleep health practice scope but repeatedly highlighted the need for targeted training for community nurses, established patient referral paths and treatment guidelines, and public health measures about sleep health as milestone interventions to fulfil such roles.

As expected in the case of Australian community nurses' patient care scope, patients with complex chronic illnesses formed a majority of the patient focus within our sample (Blay et al., 2022), and sleep disturbances and sleep disorders were therefore common issues requiring clinical management, but were often overlooked during health consultations. Our participants cited both patient attitudes or trivialisation by health professionals who prioritized more ‘pressing’ health concerns, e.g., cardiovascular or respiratory disease, as likely causative factors for this. Mounting evidence from epidemiological and experimental sleep studies highlights that prolonged periods of compromised sleep quality are associated with downstream sequelae including heightened cardiovascular disease risk, immune deficiency, mental health illness, cognitive impairment, among many other health decrements (Medic et al., 2017). Given the patient profile managed by community nurses, i.e., older patients with complex chronic diseases, sleep health quality is a modifiable risk that can improve patient's quality of life, along with other preventive health measures such as nutrition and physical activity.

It was clear that participants in the sample had good self‐reflective skills and self‐assessment regarding their sleep health awareness and clinical practice. Indeed, reiterated by most community nurse participants, inadequate sleep health education and training hindered their ability to appropriately manage sleep related presentations. This issue has been highlighted previously, with Meaklim et al. describing a clear global deficiency in sleep health education and training across all health professions, including nursing (Meaklim et al., 2020). Similar findings were observed with other health care professionals, such as GPs (suboptimal management of insomnia presentations) and pharmacists (oversupply of pharmacological sleep aids for acute insomnia concerns; Kippist et al., 2011; Sake et al., 2019). Results from surveys conducted across UK medical schools indicated minimal coverage of sleep health in UK medical curricula, resulting in lower competencies in sleep disorder diagnosis and management among medical health professionals, with little change over time (Romiszewski et al., 2020; Stores & Crawford, 1998). Health education research showcases that well‐designed educational interventions have the ability to improve clinician knowledge and patient health outcomes in both undergraduate nursing students and clinical nurse practitioners (Wu et al., 2018; Yan et al., 2022). Further, another study that conducted targeted training for nurses also demonstrated higher competence in sleep health management post training (Horii et al., 2021). This suggests that with the appropriate educational and training interventions, community nurses can be better equipped to manage sleep disturbance/disorder concerns in the community setting. Table 3 highlights potential content of targeted community nurse education/training to address gaps highlighted by participants in our study.

**TABLE 3:** Targeting sleep health knowledge and clinical expertise gaps.

Sleep health education and training proposals	Observed gaps in our study: Reasons why training may be useful
***Knowledge based aspects***—*covered through content delivery* via *lectures*, *educational videos*, *knowledge articles*, etc.	
Physiology of sleep: including sleep and wake pathways in the brain	Participants self‐expressed not having strong foundations around understanding the physiology of sleep. Understanding this aspect would help community nurses proactively explore sleep issues rather than manage issues only when reported by patients.
Understanding the need for balance between energy homeostasis (Process S) and the circadian system (Process C) for healthy sleep–wake cycles	While participants acknowledged that napping through the day can affect sleep, most participants did not understand why. Participants were also not aware of the hormonal/neurophysiological changes implicated in sleep. An understanding of physiological processes affecting sleep–wake balance could help community nurses confidently select and deliver relevant behavioural treatments to patients. It may also help community nurses tease out circadian misalignment issues (e.g., advanced sleep phase disorder) versus sleep disorders and refer appropriately.
Understanding of basic sleep pharmacology	Participants were not fully aware of how pharmacotherapeutic agents exert their effect on sleep. Understanding this would help community nurses ensure appropriateness of treatments prescribed for patients.
Available and first‐line treatments for insomnia: non‐pharmacological (e.g., behavioural therapy) and pharmacological (e.g., sedatives)	Participant did not have sufficient knowledge on first‐line behavioural treatments or on medications used for sleep. As mentioned above, understanding the principles of sedative pharmacotherapy would be useful; sedatives are often implicated in a range of adverse events in older people – thus assuring appropriate use would therefore be a key aspect of the community nurse role.
Issues with adherence	In case of behavioural treatments, the onset of action is not quick, helping patients persevere with behavioural treatments would therefore be an important issue in community nurse‐provided sleep health care. Troubleshooting or expertise with CPAP devices was a skill possessed by some participants, while CPAP adherence for OSA patients was rarely alluded to as an important aspect of sleep apnea care.
***Clinical practice aspects***—*these are skill sets best taught in practical classes, interactive workshops, role plays, teach‐back videos*, etc.	
Patient assessment skills	Most participants did not have optimal skills in sleep health information gathering – this was evident as apart from patients who complained about sleep or those receiving palliative/OSA care, sleep health assessment appeared not to be a routine part of history taking.
Use of sleep health assessment tools	Participants may not have been aware of sleep assessment tools, as such tools/questionnaires were not mentioned during the interviews. Providing participants with a toolkit for assessing specific types of sleep issues would be a pragmatic step.
Use and interpretation of sleep diaries	Only a few participants used a sleep diary with patients; however, it was unclear if they knew how to interpret the sleep diary results.
Behavioural therapy provision skills	Most participants were not aware of and did not use behavioural strategies to manage patients with insomnia. As these are now encouraged as first‐line approaches and are supported by a significant body of evidence, community nurses and all health professionals should have a key understanding of behavioural treatment principles. These ‘healthy’ sleep behaviours are important for health maintenance, not just for ‘treatment’ of sleep disorders.
Active and passive referral pathways	Participants frequently mentioned that they were not aware of patient referral points for sleep disorders/disturbance. Clearly worked pathways, referral templates, confidence in providing a referral, and facilitating referral uptake should be key skills community nurses possess. It was evident that most participants in the study were unsure of referral pathways and unclear about referral outcomes. Other health professionals should have the means to reciprocally update a referring professional about the outcome of the referral. In Australia, this could, in future, be facilitated via the electronic health record for consenting patients.
Realistic sleep health goal setting with patients	Participants were unsure about realistic sleep expectations they could convey to their patients. Patient‐directed goal setting is an important aspect of sleep health management – as behavioural change can best be driven through self‐efficacy and illness perceptions. Goal setting would also underlie ongoing management needed in most chronic conditions.

*Note*: *CPAP* Continuous positive airway pressure *OSA* Obstructive sleep apnea.

Participants reported frequently managing patient‐specific sleep disturbances, often consequent to pre‐existing comorbidities or their complications/medications, in an individualized manner. Sleep disorders such as chronic insomnia and OSA were also common presentations, and although OSA cases usually received standardized treatment (CPAP devices), insomnia was often overlooked and/or mismanaged. This phenomenon has previously been observed in the Australian primary care setting (Sake et al., 2019). While sedative and OTC/complementary medication use for insomnia has been deemed unfavourable, given the side effect profile of sedatives such as benzodiazepines and the lack of documented efficacy with OTC/complementary medicine use (Culpepper & Wingertzahn, 2015; Markota et al., 2016), this study made clear that it was a common management approach resorted to by health professionals and patients alike. When it came to non‐pharmacological management, most participants used individualized ‘sleep hygiene’ counselling; however, sleep hygiene has limited evidence supporting its use in addressing insomnia symptoms, and it has lower efficacy when used as a sole intervention without the more robustly evidenced and powerful behavioural or cognitive sleep interventions (Harvey et al., 2002; Posner & Gehrman, 2011). Indeed, in recent years, clinical insomnia experts have strongly advocated for a paradigm shift in insomnia management, promoting the provision of cognitive behavioural therapy for insomnia (CBTI) as the appropriate first‐line approach (Parliament of the Commonwealth of Australia, 2019). CBTI consists of behavioural (stimulus control, sleep restriction, and relaxation training) and cognitive interventions carried out by a sleep specialist/sleep specialized psychologist (Siebern & Manber, 2011). However, given the rising prevalence of insomnia, there is a clear demand–supply imbalance, with not enough CBTI‐specialized providers (Espie, 2009). One strategy aiming to tackle this issue was training nurses in CBTI provision (Espie et al., 2007; Torrens et al., 2021). Another strategy devised by clinical sleep experts has been to develop brief behavioural therapy for insomnia (BBTI), which is less time‐focussed than CBTI and focuses on delivering behavioural intervention components. Its effectiveness in addressing insomnia is established, and most importantly, it was devised to be delivered by nurses (Gunn et al., 2019; Kyle et al., 2020). A small‐scale randomized controlled trial conducted by Dean et al. in the United States documented the positive impact of nurse‐provided BBTI for lung cancer survivor patients with insomnia (Dean et al., 2020). Another study evaluating nurse‐provided BBTI efficacy in older age adults with chronic insomnia demonstrated positive outcomes (Buysse et al., 2011). This highlights potential for community nurses in behavioural treatment provision and can help broaden treatment accessibility by community dwelling patients living with insomnia.

Finally, factors such as time and collaborative, integrated models of patient care were frequently brought up by participants. This is not a novel issue, as community nurses in the past, both internationally and in Australia, have expressed being time‐poor and overworked (McKinless, 2020). This could lead to community nurses being unable to consult patients on their sleep health as they feel their time is limited and would need to spend it managing other comorbid health concerns. Further, enhanced collaborative treatment frameworks between community nurses and other primary health professionals could improve community sleep health provision by aiding in sleep disordered‐patient referral onto more specialized care or allowing for better communication and education on best treatment and management approaches (Franklin et al., 2015). Other tools and resources such as sleep health/disorder assessment tools, sleep diaries, and sleep health promoting mobile applications (such as those that can assist the patient with behavioural interventions for insomnia management) could also aid in improving community sleep health care provision. Nevertheless, systematic support for the community nurse profession in addressing these factors and improving current practice models is paramount. Importantly, increased sleep health‐related education and targeted training for community nurses could potentially improve their knowledge and awareness on this health topic, ultimately improving their sleep health management scope of practice. Integrating such educational resources, through curriculum courses or health professional workshops, could certainly be the way forward.

### 5.1 Strengths and limitations

While a convenience‐based snowball sampling approach may lead to sampling bias, community nurses from various practice backgrounds were approached in order to best achieve a maximally varied participant sample. Further, participants were interviewed by two separate research team members who were not previously known to any of the participants and were both from non‐nursing backgrounds. Field notes were also taken wherever applicable and discussed during team meetings. This helped decrease the influence of “interviewer” bias during interview conduction and data analysis. Importantly, the primary and senior researchers discussed data coding and thematic derivations during team meetings and thematic reviews continued until consensus was reached, alleviating “researcher bias” when analysing and presenting the data. The research team also comprised health professionals from different medical backgrounds (nursing, pharmacy and medicine), thus allowing for researcher reflexivity and a multi‐disciplinary approach to interview conduct, thematic analysis, and data presentation, mitigating “researcher subjective bias” (Dodgson, 2019).

### 5.2 Conclusion

In this qualitative study exploring Australian community nurses' sleep health practices and perspectives around care provision, we found that community nurses commonly encounter sleep disturbances/disorders in their patient cohort. Despite sleep being a frequently arising health issue, ‘gaps’ in its clinical management were evident. This was mainly due to participants' insufficient clinical expertise in this health area. Participants frequently acknowledged this and reiterated the need for increased education/training to improve their sleep health practice. Other factors, such as time to provide patient consultations and improved collaborative treatment frameworks were also regarded integral. Similar findings have been described in previous research with other primary care health professionals, highlighting the importance of future sleep health education and training for community nurses/other health practitioners and practice model reforms to overcome practice barriers to optimal sleep health care provision.

## FUNDING INFORMATION

The conduction of this research has been supported by competitive research funding from the National Health and Medical Research Council (NHMRC) of Australia, for a Centre of Research Excellence (CRE) “Positioning Primary Care at the Centre of Sleep Health Management” Application ID#1134954.

## CONFLICT OF INTEREST STATEMENT

The authors have no conflicts of interest to declare.

6

### PEER REVIEW

The peer review history for this article is available at https://publons.com/publon/10.1111/jan.15577.

## Supporting information


Appendix Table 1A

Data S1

## Data Availability

Data are available on request.

## References

[jan15577-bib-0001] Almeneessier, A. , Alamri, B. , Alzahrani, F. , Sharif, M. , Pandi‐Perumal, S. , & BaHammam, A. (2018). Insomnia in primary care settings: Still overlooked and undertreated? [original article]. Journal of Nature and Science of Medicine, 1(2), 64–68. 10.4103/jnsm.Jnsm_30_18

[jan15577-bib-0002] Ando, H. , Cousins, R. , & Young, C. (2014). Achieving saturation in thematic analysis: Development and refinement of a codebook. Comprehensive Psychology, 3(2165‐2228), Article 4. 10.2466/03.CP.3.4

[jan15577-bib-0003] Australian Primary Health Care Nurses Association . (2022). Community health nursing . Retrieved 2022 from https://www.apna.asn.au/profession/what‐is‐primary‐health‐care‐nursing/community‐health‐nursing

[jan15577-bib-0004] Bartlett, D. J. , Marshall, N. S. , Williams, A. , & Grunstein, R. R. (2008). Predictors of primary medical care consultation for sleep disorders. Sleep Medicine, 9(8), 857–864. 10.1016/j.sleep.2007.09.002 17980655

[jan15577-bib-0005] Benjafield, A. V. , Ayas, N. T. , Eastwood, P. R. , Heinzer, R. , Ip, M. S. M. , Morrell, M. J. , Nunez, C. M. , Patel, S. R. , Penzel, T. , Pépin, J. L. , Peppard, P. E. , Sinha, S. , Tufik, S. , Valentine, K. , & Malhotra, A. (2019). Estimation of the global prevalence and burden of obstructive sleep apnoea: a literature‐based analysis. The Lancet Respiratory Medicine, 7(8), 687–698. 10.1016/S2213-2600(19)30198-5 31300334 PMC7007763

[jan15577-bib-0006] Bhaskar, S. , Hemavathy, D. , & Prasad, S. (2016). Prevalence of chronic insomnia in adult patients and its correlation with medical comorbidities. Journal of Family Medicine and Primary Care, 5(4), 780–784. 10.4103/2249-4863.201153 PMC5353813 28348990

[jan15577-bib-0007] Blay, N. , Sousa, M. S. , Rowles, M. , & Murray‐Parahi, P. (2022). The community nurse in Australia. Who are they? A rapid systematic review. Journal of Nursing Management, 30(1), 154–168. 10.1111/jonm.13493 34634180 PMC9298142

[jan15577-bib-0008] Bradshaw, C. , Atkinson, S. , & Doody, O. (2017). Employing a qualitative description approach in health care research. Global Qualitative Nursing Research, 4, 2333393617742282. 10.1177/2333393617742282 29204457 PMC5703087

[jan15577-bib-0009] Braun, V. , & Clarke, V. (2006). Using thematic analysis in psychology. Qualitative Research in Psychology, 3(2), 77–101. 10.1191/1478088706qp063oa

[jan15577-bib-0010] Buysse, D. J. , Germain, A. , Moul, D. E. , Franzen, P. L. , Brar, L. K. , Fletcher, M. E. , Begley, A. , Houck, P. R. , Mazumdar, S. , Reynolds, C. F., 3rd , & Monk, T. H. (2011). Efficacy of brief behavioral treatment for chronic insomnia in older adults. International Archives of Internal Medicine, 171(10), 887–895. 10.1001/archinternmed.2010.535 PMC3101289 21263078

[jan15577-bib-0011] Chai‐Coetzer, C. L. , Antic, N. A. , Rowland, L. S. , Reed, R. L. , Esterman, A. , Catcheside, P. G. , Eckermann, S. , Vowles, N. , Williams, H. , Dunn, S. , & McEvoy, R. D. (2013). Primary care vs specialist sleep center management of obstructive sleep apnea and daytime sleepiness and quality of life: a randomized trial. Journal of the American Medical Association, 309(10), 997–1004. 10.1001/jama.2013.1823 23483174

[jan15577-bib-0012] Cheung, J. M. , Atternas, K. , Melchior, M. , Marshall, N. S. , Fois, R. A. , & Saini, B. (2014). Primary health care practitioner perspectives on the management of insomnia: A pilot study. Australian Journal of Primary Health, 20(1), 103–112. 10.1071/PY12021 24200195

[jan15577-bib-0013] Colorafi, K. J. , & Evans, B. (2016). Qualitative descriptive methods in health science research. Health Environments Research & Design, 9(4), 16–25. 10.1177/1937586715614171 PMC7586301 26791375

[jan15577-bib-0014] Colten, H. R. , & Altevogt, B. M. (2006). Sleep disorders and sleep deprivation: An unmet public health problem. In H. R. Colten & B. M. Altevogt (Eds.), Sleep disorders and sleep deprivation: An unmet public health problem. National Academies Press (US). 10.17226/11617 20669438

[jan15577-bib-0015] Culpepper, L. (2005). Insomnia: A primary care perspective. Journal of Clinical Psychiatry, 66(9), 14–17. https://www.ncbi.nlm.nih.gov/pubmed/16336037 16336037

[jan15577-bib-0016] Culpepper, L. , & Wingertzahn, M. A. (2015). Over‐the‐counter agents for the treatment of occasional disturbed sleep or transient insomnia: A systematic review of efficacy and safety. The Primary Care Companion for CNS Disorders, 17(6), 26162. 10.4088/PCC.15r01798 PMC4805417 27057416

[jan15577-bib-0017] Dean, G. E. , Weiss, C. , Jungquist, C. R. , Klimpt, M. L. , Alameri, R. , Ziegler, P. A. , Steinbrenner, L. M. , Dexter, E. U. , Dhillon, S. S. , Lucke, J. F. , & Dickerson, S. S. (2020). Nurse‐delivered brief behavioral treatment for insomnia in lung cancer survivors: A pilot RCT. Behavioral Sleep Medicine, 18(6), 774–786. 10.1080/15402002.2019.1685523 31672070 PMC7190424

[jan15577-bib-0018] DeJonckheere, M. , & Vaughn, L. M. (2019). Semistructured interviewing in primary care research: A balance of relationship and rigour. Family Medicine and Community Health, 7(2), e000057. 10.1136/fmch-2018-000057 32148704 PMC6910737

[jan15577-bib-0019] Deloitte . (2011). Re‐Awakening Australia: The Economic Cost of Sleep Disorders in Australia, 2010 . http://www.sleephealthfoundation.org.au/pdfs/news/Reawakening%20Australia.pdf

[jan15577-bib-0020] Dikeos, D. , & Georgantopoulos, G. (2011). Medical comorbidity of sleep disorders. Current Opinion in Psychiatry, 24(4), 346–354. 10.1097/YCO.0b013e3283473375 21587079

[jan15577-bib-0021] Dodgson, J. E. (2019). Reflexivity in qualitative research. Journal of Human Lactation, 35(2), 220–222. 10.1177/0890334419830990 30849272

[jan15577-bib-0022] Doyle, L. , McCabe, C. , Keogh, B. , Brady, A. , & McCann, M. (2020). An overview of the qualitative descriptive design within nursing research. Journal of Research in Nursing, 25(5), 443–455. 10.1177/1744987119880234 34394658 PMC7932381

[jan15577-bib-0023] Espie, C. A. (2009). “Stepped care”: A health technology solution for delivering cognitive behavioral therapy as a first line insomnia treatment. Sleep, 32(12), 1549–1558. 10.1093/sleep/32.12.1549 20041590 PMC2786038

[jan15577-bib-0024] Espie, C. A. , MacMahon, K. M. , Kelly, H. L. , Broomfield, N. M. , Douglas, N. J. , Engleman, H. M. , McKinstry, B. , Morin, C. M. , Walker, A. , & Wilson, P. (2007). Randomized clinical effectiveness trial of nurse‐administered small‐group cognitive behavior therapy for persistent insomnia in general practice. Sleep, 30(5), 574–584. 10.1093/sleep/30.5.574 17552372

[jan15577-bib-0025] Franklin, C. M. , Bernhardt, J. M. , Lopez, R. P. , Long‐Middleton, E. R. , & Davis, S. (2015). Interprofessional teamwork and collaboration between community health workers and healthcare teams: An integrative review. Health Services Research and Managerial Epidemiology, 2, 2333392815573312. 10.1177/2333392815573312 28462254 PMC5266454

[jan15577-bib-0026] Gunn, H. E. , Tutek, J. , & Buysse, D. J. (2019). Brief behavioral treatment of insomnia. Sleep Medicine Clinics, 14(2), 235–243. 10.1016/j.jsmc.2019.02.003 31029189

[jan15577-bib-0027] Hale, L. , Troxel, W. , & Buysse, D. J. (2020). Sleep health: An opportunity for public health to address health equity. Annual Review of Public Health, 41, 81–99. 10.1146/annurev-publhealth-040119-094412 PMC7944938 31900098

[jan15577-bib-0028] Hamilton, S. G. , & Joosten, S. A. (2017). Obstructive sleep apnoea and obesity. Australian Journal for General Practitioners, 46, 460–463 https://www.racgp.org.au/afp/2017/july/obstructive‐sleep‐apnoea‐and‐obesity 28697288

[jan15577-bib-0029] Hanes, C. A. , Wong, K. K. , & Saini, B. (2015). Consolidating innovative practice models: The case for obstructive sleep apnea services in Australian pharmacies. Research in Social and Administrative Pharmacy, 11(3), 412–427. 10.1016/j.sapharm.2014.08.009 25262387

[jan15577-bib-0030] Harvey, L. , Inglis, S. J. , & Espie, C. A. (2002). Insomniacs' reported use of CBT components and relationship to long‐term clinical outcome. Behaviour Research and Therapy, 40(1), 75–83. 10.1016/s0005-7967(01)00004-3 11762429

[jan15577-bib-0031] Horii, S. , Nguyen, C. T. M. , Pham, H. T. T. , Amaike, N. , Ho, H. T. , & Aiga, H. (2021). Effectiveness of a standard clinical training program in new graduate nurses' competencies in Vietnam: A quasi‐experimental longitudinal study with a difference‐in‐differences design. PLoS ONE, 16(7), e0254238. 10.1371/journal.pone.0254238 34242294 PMC8270421

[jan15577-bib-0032] Kerkstra, A. , & Vorst‐Thijssen, T. (1991). Factors related to the use of community nursing services in The Netherlands. Journal of Advanced Nursing, 16(1), 47–54. 10.1111/j.1365-2648.1991.tb01496.x 2005289

[jan15577-bib-0033] Khandelwal, D. , Dutta, D. , Chittawar, S. , & Kalra, S. (2017). Sleep Disorders in Type 2 Diabetes. Indian Journal of Endocrinology & Metabolism, 21(5), 758–761. 10.4103/ijem.IJEM_156_17 28989888 PMC5628550

[jan15577-bib-0034] Kippist, C. , Wong, K. , Bartlett, D. , & Saini, B. (2011). How do pharmacists respond to complaints of acute insomnia? A simulated patient study. International Journal of Clinical Pharmacy, 33(2), 237–245. 10.1007/s11096-011-9482-5 21394573

[jan15577-bib-0035] Kyle, S. D. , Madigan, C. , Begum, N. , Abel, L. , Armstrong, S. , Aveyard, P. , Bower, P. , Ogburn, E. , Siriwardena, A. , Yu, L. M. , & Espie, C. A. (2020). Primary care treatment of insomnia: study protocol for a pragmatic, multicentre, randomised controlled trial comparing nurse‐delivered sleep restriction therapy to sleep hygiene(the HABIT trial). British Medical Journal Open, 10(3), e036248. 10.1136/bmjopen-2019-036248 PMC7059413 32139496

[jan15577-bib-0036] LeBlanc, E. S. , Smith, N. X. , Nichols, G. A. , Allison, M. J. , & Clarke, G. N. (2018). Insomnia is associated with an increased risk of type 2 diabetes in the clinical setting. British Medical Journal Open Diabetes Research & Care, 6(1), e000604. 10.1136/bmjdrc-2018-000604 PMC6326337 30687505

[jan15577-bib-0037] Lin, C. L. , Chien, W. C. , Chung, C. H. , & Wu, F. L. (2018). Risk of type 2 diabetes in patients with insomnia: A population‐based historical cohort study. Diabetes/Metabolism Research and Reviews, 34(1), e2930. 10.1002/dmrr.2930 28834008

[jan15577-bib-0038] Mackenzie, N. , & Knipe, S. (2006). Research dilemmas: Paradigms, methods and methodology. Issues in Educational Research, 16(2), 193–205.

[jan15577-bib-0039] Markota, M. , Rummans, T. A. , Bostwick, J. M. , & Lapid, M. I. (2016). Benzodiazepine Use in Older Adults: Dangers, Management, and Alternative Therapies. Mayo Clinic Proceedings, 91(11), 1632–1639. 10.1016/j.mayocp.2016.07.024 27814838

[jan15577-bib-0040] McKinless, E. (2020). Impact of stress on nurses working in the district nursing service. The British Journal of Community Nursing, 25(11), 555–561. 10.12968/bjcn.2020.25.11.555 33161741

[jan15577-bib-0041] Meaklim, H. , Jackson, M. L. , Bartlett, D. , Saini, B. , Falloon, K. , Junge, M. , Slater, J. , Rehm, I. C. , & Meltzer, L. J. (2020). Sleep education for healthcare providers: Addressing deficient sleep in Australia and New Zealand. Sleep Health, 6(5), 636–650. 10.1016/j.sleh.2020.01.012 32423774

[jan15577-bib-0042] Medic, G. , Wille, M. , & Hemels, M. E. (2017). Short‐ and long‐term health consequences of sleep disruption. Nature and Science of Sleep, 9, 151–161. 10.2147/NSS.S134864 PMC5449130 28579842

[jan15577-bib-0043] Motamedi, K. K. , McClary, A. C. , & Amedee, R. G. (2009). Obstructive sleep apnea: A growing problem. The Ochsner Journal, 9(3), 149–153 https://www.ncbi.nlm.nih.gov/pubmed/21603432 21603432 PMC3096276

[jan15577-bib-0044] Neel, A. B., Jr. (2013). 10 Types of Meds That Can Cause Insomnia, Accessed 2020 . https://www.aarp.org/health/drugs‐supplements/info‐04‐2013/medications‐that‐can‐cause‐insomnia.html

[jan15577-bib-0045] Nowell, S. L. , Norris, J. M. , White, D. E. , & Moules, N. J. (2017). Thematic analysis: Striving to meet the trustworthiness criteria. International Journal of Qualitative Methods, 16(1), 160940691773384. 10.1177/1609406917733847

[jan15577-bib-0046] Parker, C. , Scott, S. , & Geddes, A. (2019). Snowball sampling. SAGE Research Methods Foundations https://uk.sagepub.com/en¬gb/eur/srm¬foundations

[jan15577-bib-0047] Parliament of the Commonwealth of Australia . (2019). Bedtime Reading ‐ parliamentary inquiry report . https://www.sleephealthfoundation.org.au/news/special‐reports/bedtime‐report‐parliamentary‐inquiry‐report.html

[jan15577-bib-0048] Posner, D. , & Gehrman, P. R. (2011). Chapter 3 ‐ Sleep hygiene. In M. Perlis , M. Aloia , & B. Kuhn (Eds.), Behavioral treatments for sleep disorders (pp. 31–43). Academic Press. 10.1016/B978-0-12-381522-4.00003-1

[jan15577-bib-0049] Romiszewski, S. , May, F. E. K. , Homan, E. J. , Norris, B. , Miller, M. A. , & Zeman, A. (2020). Medical student education in sleep and its disorders is still meagre 20 years on: A cross‐sectional survey of UK undergraduate medical education. Journal of Sleep Research, 29(6), e12980. 10.1111/jsr.12980 32166824

[jan15577-bib-0050] Rosala, M. , & Moran, K. (2022). The funnel technique in qualitative user research. Nielsen Norman Group. https://www.nngroup.com/articles/the‐funnel‐technique‐in‐qualitative‐user‐research/

[jan15577-bib-0051] Rosenberg, R. P. (2021). The bidirectional relationship between insomnia and comorbid disorders. Journal of Clinical Psychiatry, 82(2), EI20008BR20002C. 10.4088/jcp.Ei20008br2c 33989460

[jan15577-bib-0052] Sake, F. T. , Wong, K. , Bartlett, D. J. , & Saini, B. (2018). Integrated primary care insomnia management in Australia. Research in Social and Administrative Pharmacy, 14(2), 170–179. 10.1016/j.sapharm.2017.02.013 28262515

[jan15577-bib-0053] Sake, F. T. , Wong, K. , Bartlett, D. J. , & Saini, B. (2019). Insomnia Management in the Australian Primary Care Setting. Behavioral Sleep Medicine, 17(1), 19–30. 10.1080/15402002.2016.1266491 28098492

[jan15577-bib-0054] Sandelowski, M. (2010). What's in a name? Qualitative description revisited. Research in Nursing & Health, 33(1), 77–84. 10.1002/nur.20362 20014004

[jan15577-bib-0055] Scott, A. J. , Webb, T. L. , Martyn‐St James, M. , Rowse, G. , & Weich, S. (2021). Improving sleep quality leads to better mental health: A meta‐analysis of randomised controlled trials. Sleep Medicine Reviews, 60, 101556. 10.1016/j.smrv.2021.101556 34607184 PMC8651630

[jan15577-bib-0056] Siebern, A. T. , & Manber, R. (2011). New developments in cognitive behavioral therapy as the first‐line treatment of insomnia. Psychology Research and Behavior Management, 4, 21–28. 10.2147/PRBM.S10041 22114532 PMC3218784

[jan15577-bib-0057] Stores, G. , & Crawford, C. (1998). Medical student education in sleep and its disorders. Journal of the Royal College of Physicians of London, 32(2), 149–153 https://www.ncbi.nlm.nih.gov/pubmed/9597633 9597633 PMC9663016

[jan15577-bib-0058] Tong, A. , Sainsbury, P. , & Craig, J. (2007). Consolidated criteria for reporting qualitative research (COREQ): A 32‐item checklist for interviews and focus groups. International Journal for Quality in Health Care, 19(6), 349–357. 10.1093/intqhc/mzm042 17872937

[jan15577-bib-0059] Torrens, I. , Esteva, M. , Vicens, C. , Pizá‐Portell, M. R. , Vidal‐Thomàs, M. C. , Vidal‐Ribas, C. , Lorente‐Montalvo, P. , & Torres‐Solera, E. (2021). Assessing the feasibility and acceptability of a cluster‐randomized study of cognitive behavioral therapy for chronic insomnia in a primary care setting. BMC Family Practice, 22(1), 77. 10.1186/s12875-021-01429-5 33863276 PMC8052716

[jan15577-bib-0060] Worley, S. L. (2018). The extraordinary importance of sleep: The detrimental effects of inadequate sleep on health and public safety drive an explosion of sleep research. P & T: A Peer‐Reviewed Journal for Formulary Management, 43(12), 758–763 https://pubmed.ncbi.nlm.nih.gov/30559589 30559589 PMC6281147

[jan15577-bib-0061] Wu, Y. , Brettle, A. , Zhou, C. , Ou, J. , Wang, Y. , & Wang, S. (2018). Do educational interventions aimed at nurses to support the implementation of evidence‐based practice improve patient outcomes? A systematic review. Nurse Education Today, 70, 109–114. 10.1016/j.nedt.2018.08.026 30179782

[jan15577-bib-0062] Yan, Z. , Chang, H.‐C. , Montayre, J. , & Ho, M.‐H. (2022). How does geriatric nursing education program change the knowledge, attitude and working intention among undergraduate nursing students? A systematic literature review. Nurse Education Today, 108, 105161. 10.1016/j.nedt.2021.105161 34649069

[jan15577-bib-0063] Yeghiazarians, Y. , Jneid, H. , Tietjens, J. R. , Redline, S. , Brown, D. L. , El‐Sherif, N. , Mehra, R. , Bozkurt, B. , Ndumele, C. E. , & Somers, V. K. (2021). Obstructive Sleep Apnea and Cardiovascular Disease: A Scientific Statement From the American Heart Association. Circulation, 144(3), e56–e67 https://www.ahajournals.org/doi/10.1161/CIR.0000000000000988 34148375 10.1161/CIR.0000000000000988

