# Supplementary material for: Improving sleep health management in primary care: A potential role for community nurses?
Source: J Adv Nurs. 2023 Feb 9;79(6):2236–49. doi: 10.1111/jan.15577 (PMC10952398; doi:10.1111/jan.15577)
Supplement: Supplementary file 1 — Appendix Table 1A [file JAN-79-2236-s001.docx]

**Appendix**

**Table 1A:** Semi-structured interview guide for community nurses

| Interview questions | Prompts to be used only if needed | Aim of the question |
| --- | --- | --- |
| **I would like to start by asking what the term ‘sleep disorders’ generally implies to you?**  **To follow on, similarly may I ask what the term ‘circadian rhythm disorders’ generally implies to you?** | Not applicable | This question aims to understand what participants view, define or understand sleep/circadian disorders to be |
| **Next, may I ask you to describe the general extent and type of sleep disorders you see in your scope of practice?** | - Presentations Frequency (e.g., weekly) - Different complaint types / (explore most common). - Diagnosed/undiagnosed - ‘at risk’ populations (explore) | - This question aims to elicit the level/pattern/experience of sleep disorder involvement of the participant |
| **How do discussions about sleep health in your practice setting usually occur?** | - Referral by doctor/GP/pharmacist/other - Patient initiated/initiated by you - During discussions about lifestyle - During care plans or formal assessments | Often sleep health discussions are reactive – initiated by patient. This question seeks to understand how discussions about sleep health evolve in primary/community care nursing practice |
| **Would you please describe your involvement in the care of people with sleep disorders in your practice?** | - Explore level of involvement - Barriers to involvement - Enablers to involvement | This question aims to understand how community nurses are involved in sleep health management |
| ***NEXT QUESTIONS TO BE SHAPED BY ANSWERS TO ABOVE QUESTIONS*** | | |
| **For the most common types of presentations, what general approach do you use to assess the problem?**  *Let’s discuss this for the two most common disorders you encounter – for e.g., insomnia and apnea* | - Questionnaires/tools (explore) - Apps/Diaries - Medication history/review - Symptom history - Different assessments for diagnosed vs undiagnosed | This question aims to understand how community nurses assess sleep problems |
| **For the most common types of presentations, what general approach do you use to provide care for patients with sleep disorders?**  *Let’s discuss this for the two most common disorders you see in practice.* | - Referral - Counselling about sleep hygiene - Behavioural Treatments (explore) - Medication/product/device recommendation (explore) - CPAP Education and services | This question aims to understand how community nurses manage sleep problems |
| **How confident do you feel in helping patients with sleep and circadian disorders? And why?** | - Explore reasons for confidence/non-confidence | This question aims to gauge the level of confidence community nurses have in managing sleep disorders |
| **In general, what roles do you think nurses (community nurses/practice nurses) can play in sleep health?** | - Screening - Patient education - Behavioural treatment provision - Medication/product recommendation - CPAP provision and monitoring - Referral | This question aims to find out what nurse practitioners think their role in sleep health is |
| **How can nurses roles be better integrated with general primary care for improved patient with sleep disorders management?** | - General Practice Nurses - Community nurses - Clear referral pathways (GPs/Psychologists) - Remuneration/practice incentives - Working with other professionals in community | This question prompts participants to consider how they can work in integrated care models |
| **In your opinion, what are the key barriers to improved sleep health management in current primary care practice?** | - Health professional awareness - Public awareness - Time/work-pressures - Patient attitude - Inappropriate prescribing - Advertising of products | This question aims to find from the participants viewpoint what they perceive as barriers to sleep health/sleep disorder management |
| **If some practice resources were being developed to improve sleep health management by nurses, what resources would you require?** | - Clear guidelines - Training (probe preferred training modes) - Structured pathways - Pathways for service remuneration | This question aims to elicit a ‘wish-list’ of resources that practitioners feel would improve current practice. |
